# Importance of Native Grassland Habitat for Den-Site Selection of Indian Foxes in a Fragmented Landscape

**DOI:** 10.1371/journal.pone.0076410

**Published:** 2013-10-03

**Authors:** Girish Arjun Punjabi, Ravi Chellam, Abi Tamim Vanak

**Affiliations:** 1 Post Graduate Programme in Wildlife Biology and Conservation, Wildlife Conservation Society - India Program, National Centre for Biological Sciences, Tata Institute of Fundamental Research, Bengaluru, India; 2 Madras Crocodile Bank Trust/ Centre for Herpetology, Mamallapuram, Tamil Nadu, India; 3 Centre for Biodiversity and Conservation, Ashoka Trust for Research in Ecology and the Environment, Bengaluru, India; 4 School of Life Sciences, University of KwaZulu-Natal, Westville, Durban, South Africa; The Australian National University, Australia

## Abstract

Fragmentation of native habitats is now a ubiquitous phenomenon affecting wildlife at various scales. We examined selection of den-sites (n = 26) by Indian foxes (*Vulpes bengalensis*) in a highly modified short-grassland landscape in central India (Jan-May, 2010). At the scale of the home-range, defined by an 800 m circular buffer around den sites, we examined the effect of land-cover edges and roads on selection of sites for denning using a distance-based approach. At the smaller den-area scale, defined by a 25 m x 25 m plot around den and paired available sites, the effect of microhabitat characteristics was examined using discrete-choice models. Indian foxes selected den-sites closer to native grasslands (t = -9.57, P < 0.001) and roads (t = -2.04, P = 0.05) than random at the home-range scale. At the smaller scale, abundance of rodents and higher visibility increased the odds of selection of a site by eight and four times respectively, indicating resource availability and predator avoidance to be important considerations for foxes. Indian foxes largely chose to den in human-made structures, indicated by the proportion of dens found in earthen bunds (0.69) and boulder piles (0.27) in the study area. With agricultural expansion and human modification threatening native short-grassland habitats, their conservation and effective management in human-dominated landscapes will benefit the Indian fox. The presence of some human-made structures within native grasslands would also be beneficial for this den-dependent species. We suggest future studies examine the impact of fragmentation and connectivity of grasslands on survival and reproductive success of the Indian fox.

## Introduction

Habitat modification and fragmentation are recognized globally as important drivers of biodiversity loss [1,2]. Human modification and fragmentation create landscape heterogeneity in native habitats and may threaten species by deterministic and stochastic processes [2]. Studies indicate that species’ responses to human modification are dependent upon the ecology of the species involved, the amount of habitat change, and the rate at which species adapt to changes in their environments [3-6]. In India, the tropical short grassland plains [7] are a fragmented mosaic of native grasslands and human-modified areas. Short grassland ecosystems across the country are only a peripheral focus of conservation attention, and there has been considerable fragmentation and alteration of these grasslands through intense agriculture and industrialization [7]. Despite this, few studies have quantitatively assessed species’ responses to modification in such systems [8].

The Indian fox (*Vulpes bengalensis*) is a small-sized canid, endemic to the Indian sub-continent, and found in semi-arid, flat to undulating terrain, including tropical short grasslands [9]. The species is currently listed as Least Concern in the IUCN red list [10]. It is widely distributed, but current trends indicate that populations of the species are declining [10]. This is largely attributed to the loss of habitat, and recent studies demonstrate that the spatial ecology of the species is heavily influenced by the presence of native grasslands [8,11]. The Indian fox is a den-dependent species, and breeding pairs use dens primarily for reproduction and pup-rearing [12,13]. Dens are also used for resting, but such use is limited to the peak of the dry season (April-May) [8]. Indian foxes may use more than one breeding den during the reproductive period, possibly as a strategy to avoid predators [13]. Thus, availability of suitable sites for denning would eventually affect reproductive success of the species.

We examined patterns of den selection by Indian foxes in a human-modified, short grassland landscape at two scales. At the larger ‘home-range’ scale (similar to Johnson’s third-order habitat selection [14]) we used a Euclidian distance-based approach [15] to examine the effect of dominant land cover edges and roads on selection of den-sites. Based on available literature [8,10,12,16], and our observations, we expected Indian foxes to den closer to native grassland habitats than random. Foxes were also expected to den farther away from human-dominated areas such as agricultural-land, built-up areas (human-occupied areas), and roads than random.

We then examined the effect of microhabitat features at the smaller ‘den-area’ scale (in the near vicinity of the den) using a case-control design. For microhabitat characteristics, we examined the effect of den substrate, visibility, shrub density, and rodent burrows by using discrete choice models [17]. In case of den substrate, previous studies in the area [18,19] suggested the presence of human-made structures such as earthen bunds or boulder piles (canal or well tailings and mounds) positively influence selection of den sites. Earthen bunds are made for water and soil conservation, as well as to demarcate individual fields, while boulder piles result from digging activities for irrigation. We also expected sites with higher visibility to improve detection of predators, which include free-ranging dogs (*Canis familiaris*), that are known to be major interference competitors of Indian foxes in this landscape [20]. Lastly, small rodents (body length ≤ 20 cm, weight = *c.* 200 g) and wild fruit (viz. *Zizyphus mauritiana* and *Cássia* sp.) are significant components of Indian fox diet [21,22]; therefore we expected higher abundance of small rodents (*Tatera indica*, *Golunda ellioti*, and *Mus* sp.) and wild fruit to positively influence selection of den-sites at the smaller scale.

The overall aim of the study was to examine the denning ecology of a lesser-known wild canid in a fragmented and human-modified short-grassland landscape. Our species-specific analysis can be valuable for management of this species, which is often overlooked in conservation decision-making regarding threatened grassland biomes of India.

## Methods

### Ethics statement

The study was conducted under the permission of the Maharashtra State Forest Department (Permit no. D-22(8)/Research/3416/2009-10) on the Indian fox (*V. bengalensis*) listed in Schedule II of the Indian Wildlife (Protection) Act, 1972. Since this was a purely observational study, no animal care and use committee approval was required.

**Figure 1:**
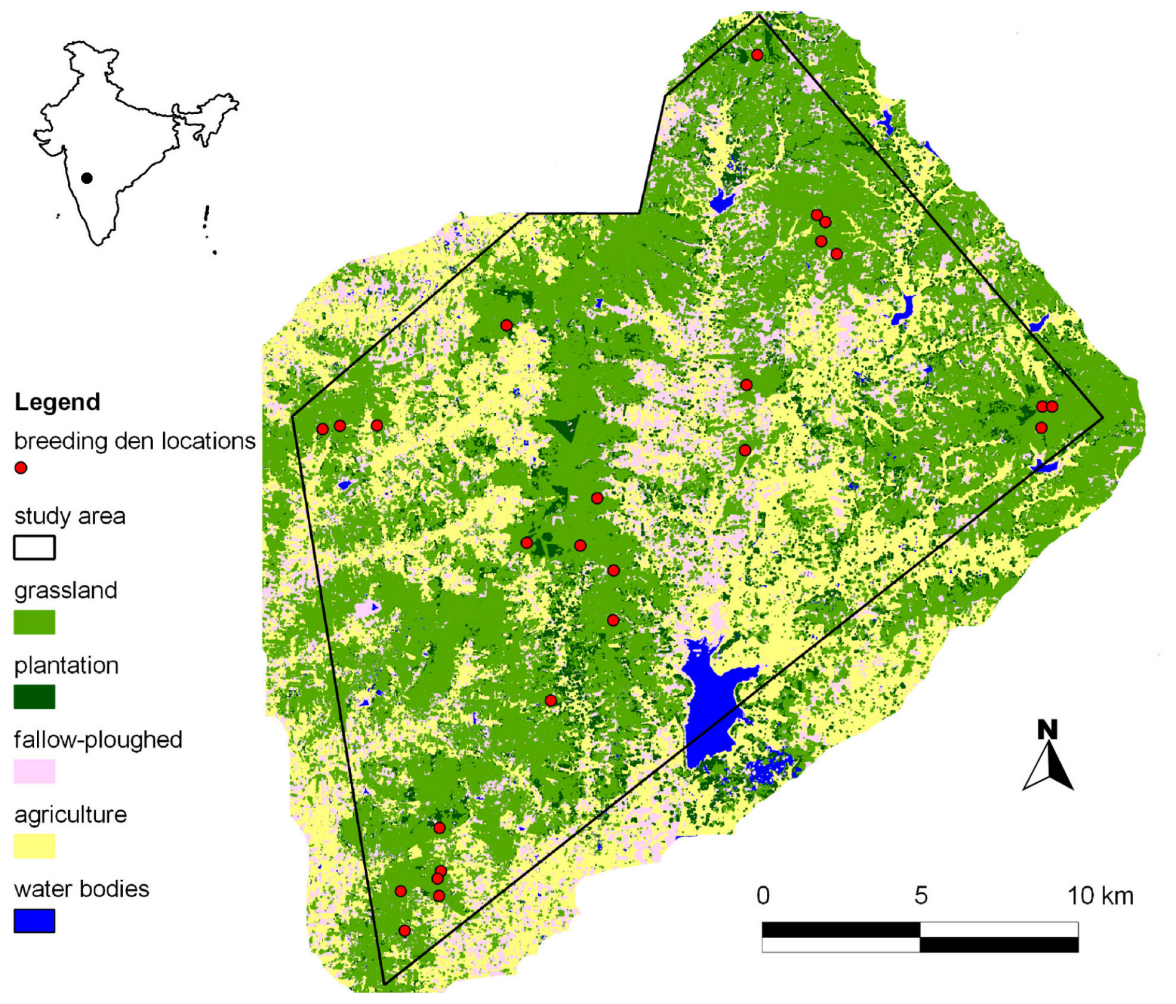
Study area map. The map shows the *c*. 460 km^2^ study area with breeding den locations of Indian foxes (*V. bengalensis*) and dominant land-cover categories in and around the Great Indian Bustard Sanctuary in 2009-10.

We conducted this study in and around the Great Indian Bustard Sanctuary (GIBS) near Nannaj village, Maharashtra (17°49’40’’ N and 75°51’35’’ E, WGS 84 datum) in Central India from January to May 2010. We defined this study area by creating a minimum convex polygon around active fox dens and available sites, buffered by 800m radius sampling distance (see Figure 1 for study area map). The total study area of *c.* 460 km^2^ comprised portions of the Great Indian Bustard sanctuary falling in Solapur and Osmanabad districts of Maharashtra state. We had limited access to six protected grassland and plantation patches (*c*. six km^2^), as these were the protected habitat enclaves for the endangered great Indian bustard (*Ardeotis nigriceps*). However, previous studies found relatively few breeding dens in these protected patches (<5) (A.T. Vanak, personal observation), hence excluding these areas from sampling is unlikely to have had a major influence on the results of this study.

The study region experiences distinct seasonal variations, with most of the precipitation received during July-October (annual precipitation = 600 mm, temp = 16-32 °C) [18]. Annual temperatures vary from 6°C in the cool-dry season (November-February) to 47°C in the hot-dry season (March-June) [18]. Human population densities vary from 176/km^2^ to 1305/km^2^ in areas close to Solapur city (http://solapur.gov.in/). The region is a mixed-use landscape dominated by short grasslands, which included protected grassland patches, and communal and private grazing lands. Short grassland areas resemble savanna habitats, being dominated by *Sehima-Dicanthium* grasses [7], intermixed with forbs and occasionally small trees. Forestry plantations comprised of Neem (*Azadirachta indica*), Babul (*Acacia* sp.), Mexican lilac (*Glyricidia sepium*), and Eucalyptus (*Eucalyptus* sp.), and are mostly owned by the Maharashtra State Forest Department. Ploughed-land, fallow-land, and agricultural-land were other land cover categories in the area. Agricultural-land was dominated by dry-zone crops, and included irrigated vineyards and pomegranate orchards within the agricultural matrix. Agricultural areas in the region are normally unfenced, which makes them potentially available for wildlife to use. The remaining area consisted of villages and buildings (built-up areas), water bodies, and roads.

### Den surveys

We initially checked the status of known den locations (n = 31) from previous studies in the region [18,19]. We then conducted systematic walking searches in the region with two to three observers looking for active Indian fox breeding den-sites. Approximately 760 hours were spent searching for Indian fox dens over four months (January to April) in the area. We also opportunistically made inquiries with farmers and shepherds about locations of den sites. We spent about 60% of the walk effort surveying grasslands (occupying *c.* 25% of the study area) and plantations (occupying *c.* 22% of the study area). The rest of the survey effort was spent in surveying agricultural fields and edges (*c.* 24% of the study area), fallow-lands (*c.* 12% of the study area), and ploughed lands (*c.* 10% of the study area). Based on a combination of prior knowledge of den-sites from radio telemetry, and our exhaustive surveys of the study area, we are confident that we detected most breeding dens and that our survey effort was not unduly biased towards any habitat type.

Active dens (in-use during the study period) were identified by signs of fresh excavation, pup and adult scat deposition around the den-site, and tracks along the ramp of the den openings. Since Indian foxes use dens for two purposes, we differentiated between active breeding and resting dens by the presence of scats of pups and pup tracks around the den-site. On most occasions, this was reconfirmed by noting the presence of pups outside the dens during dawn and dusk. We considered both dens, natal and non-natal, as breeding dens, in case we found evidence of den shifting. Resting dens were not considered for analysis, as they are used by adults mostly during the peak of the dry season (April-May).

### Selection of scales

We used a Euclidian distance-based approach [15], to examine the impact of dominant land cover edges and roads on selection of den-sites within the home-range scale. When compared to the classification-based approach, the distance-based approach has advantages in detecting the importance of edges, and flexibility in using polygon, linear, as well as point features in the analysis [23].

We defined the home-range scale by using a distance of 800 m to create a buffer around each den site. This distance was chosen since it comprised an area of *c.* 200 ha, which is about midway between documented annual home range sizes of male (307.9 ha ± 48SE), and female (164.7 ha ± 37.8SE) Indian foxes [8]. Due to the lack of collared animals, we did not use exact home-range sizes of denning pairs in this study; however, we feel that the 800 m buffer radius adequately represented most of the actual home-ranges of denning foxes.

We used a uniform distribution to generate 200 random points in each den buffer (about 100 points/ km^2^). All analyses were conducted in Quantum GIS (1.8.0 Lisboa) [24]. We calculated distances to nearest representative feature of land cover types and roads for den and random points in GRASS [25], using a land cover map of the area. We created this supervised land-cover map using high resolution (5.8 m) multi-spectral images obtained from the Indian Remote Sensing Satellite (IRS-P6, National Remote Sensing Agency, Hyderabad) for the months of December 2008 and February 2009. Signatures were collected using ground truth data and high resolution Google, Earth imagery [26] of the study site. We did not formally assess accuracy; however, we compared the final map with Google, Earth imagery of the study site to ensure no major misclassification existed.

A distance of zero was considered for the land cover type that contained the den. A total of seven distance ratios were created for each den (six land cover types and one for roads), where a ratio comprised the nearest distance from the den to each land cover or road feature, divided by the average distance of all random points for every den to these features. To examine whether Indian foxes exhibited habitat selection (or avoidance) when choosing den sites as opposed to random, we used a MANOVA to test if the mean of the vector of distance ratios differed from a vector of 1s [15]. If the MANOVA was significant, we compared means of the distance ratios using bootstrapping (1000 times), to determine if they differed from one. Distance ratios less than one indicated selection of the feature, and ratios greater than one indicated avoidance of the feature [15]. We also performed paired t-tests to corroborate results from bootstrapping. Relative use of different land cover types and roads were then determined using paired t-tests across them.

At the den-area scale, we recorded microhabitat characteristics in close vicinity of the den-site. We examined characteristics using a 25 m X 25 m north oriented plot with the den-site as the centre. We measured the same variables at four putatively available points at a distance of 300 m in four cardinal directions from the den-site (as per [27]). We examined the effect of den substrate, visibility, shrub density, and rodent abundance at the den-area scale.

Den substrate at a site was recorded and classified either as an ‘earthen bund’, ‘boulder pile’ (in case of rocky mounds, or canal and well tailings), or ‘other’ category (if no artificial substrates were present). Visibility at each den and available site was measured with a Robel pole [28] at a distance of 10 m from the centre of the site in eight directions. These values were averaged for each site, and ranged between 0-15, indicating low to high visibility. As an index of fruit abundance, we ascertained shrub density by counting the number of shrubs in the 25 m X 25 m plots at the used and paired available sites. In the study area, shrubs are primarily dominated by species of *Zizyphus* and *Cássia*. Although *Zizyphus* is a stunted tree (

< 3 m), most trees of the species we encountered were small (< 1 m) and of homogenous size due to heavy lopping, therefore we included it as a shrub for the purpose of the study. Lastly, we quantified the abundance of small rodents, the principal prey group, at the den-area scale by counting the number of rodent burrows within each square plot around used and paired available sites. Active burrows counts correlate well with actual rodent abundance [29], and are a feasible index of small rodent abundance [20].

We tested for possible multicollinearity among variables by calculating variance inflation factor (VIF) values. We standardized all continuous variables by dividing each value of the variable by its sample mean and standard deviation [30] to avoid potential model convergence problems. Den substrate was used as a categorical variable with 3 factor levels (one for each type). Each used point (den site) was paired with four available points (available sites) in the discrete choice analysis [17]. We used an information theoretic approach of testing models (variables and interactions) using Akaike’s information criterion corrected for small sample sizes (AICc) and assessed model weights (*w*_*i*_) [31]. We averaged the variables of top models (Δ AICc ≤ 4), since they adequately represented the confidence set [32]. We performed k-fold cross validations to evaluate the predictive ability of the top models [33]. All analyses were conducted in R version 2.15.2 [34].

## Results

We found a total of 26 active Indian fox breeding dens in the study area from January to April 2010. Most dens (19 dens) occurred in grasslands, followed by fallow-land (3 dens), agricultural fields (2 dens), and plantations and ploughed land (one den each).

**Figure 2:**
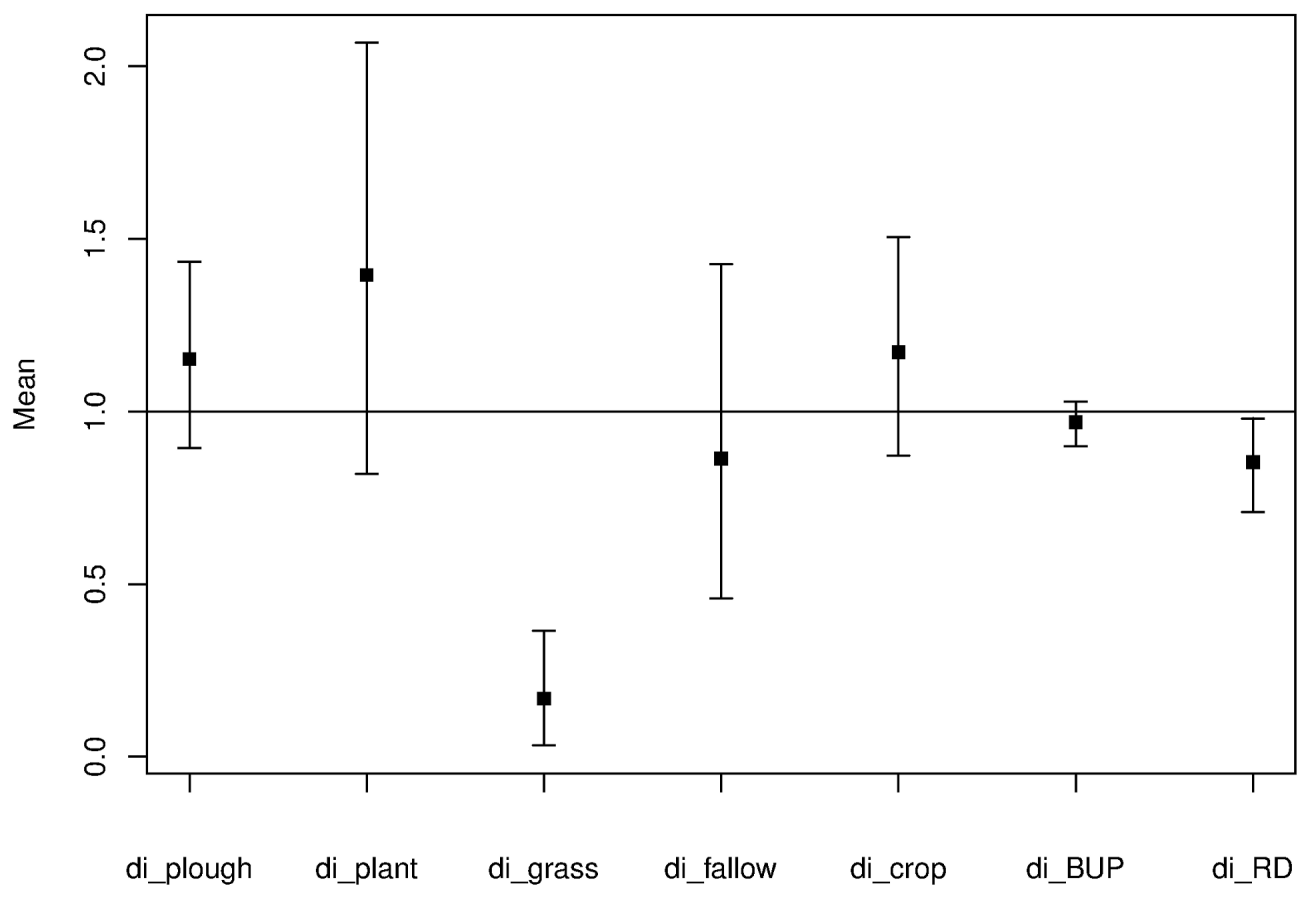
Results of Euclidian distance analysis at the home-range scale. Figure shows the mean of distance ratios of habitat types (indicated as di_habitat type) with 95% confidence intervals as compared to 1. Values below 1 indicate selection, while values above 1 indicate avoidance of habitat types at the home-range scale.

At the home-range scale, Indian foxes exhibited habitat selection at den sites (Pillai’s Trace = 0.98, F = 123.71 and P < 0.001). Mean of distance ratios and results of 95% confidence intervals using bootstrap indicated den sites to be closer to grasslands (t = -9.57, P < 0.001) and roads (t = -2.04, P = 0.05) than random points (Figure 2). Foxes showed no significant selection or avoidance for other land cover types (plantations and agricultural, fallow, ploughed and built-up lands) (Figure 2, Table 1). Relative rankings indicated that grasslands were selected significantly over other land cover types at the home-range scale (Table 1).

**Table 1:** Table shows test statistic (t) and significance values from paired t-tests comparing mean of distance ratios of land cover types with a vector of 1s, and relative rankings of the variables.

**^a^Variable**	**^b^Ratio**	**t**	**Sig (≤ 0.05)**	**^c^Rank**
plough	1.15	1.09	0.29	B
plant	1.39	1.19	0.25	B
**grass**	**0.16**	**-9.57**	<0.001*	**A**
fallow	0.86	-0.55	0.59	B
crop	1.71	1.04	0.31	B
built-up	0.97	-0.92	0.37	B
**road**	**0.85**	**-2.04**	0.05*	B

a plough = ploughed-land, plant = plantation, grass = grassland, fallow = fallow-land, crop = agricultural-land, built-up = built-up areas, road = roads

b Ratio = Mean of distance ratios of land cover types and roads

c Rank = Relative ranking of variables based on paired t-tests between variables, same letters indicate no significant difference (P > 0.05) in preference among variables

* indicates a significant difference from 1

**Figure 3:**
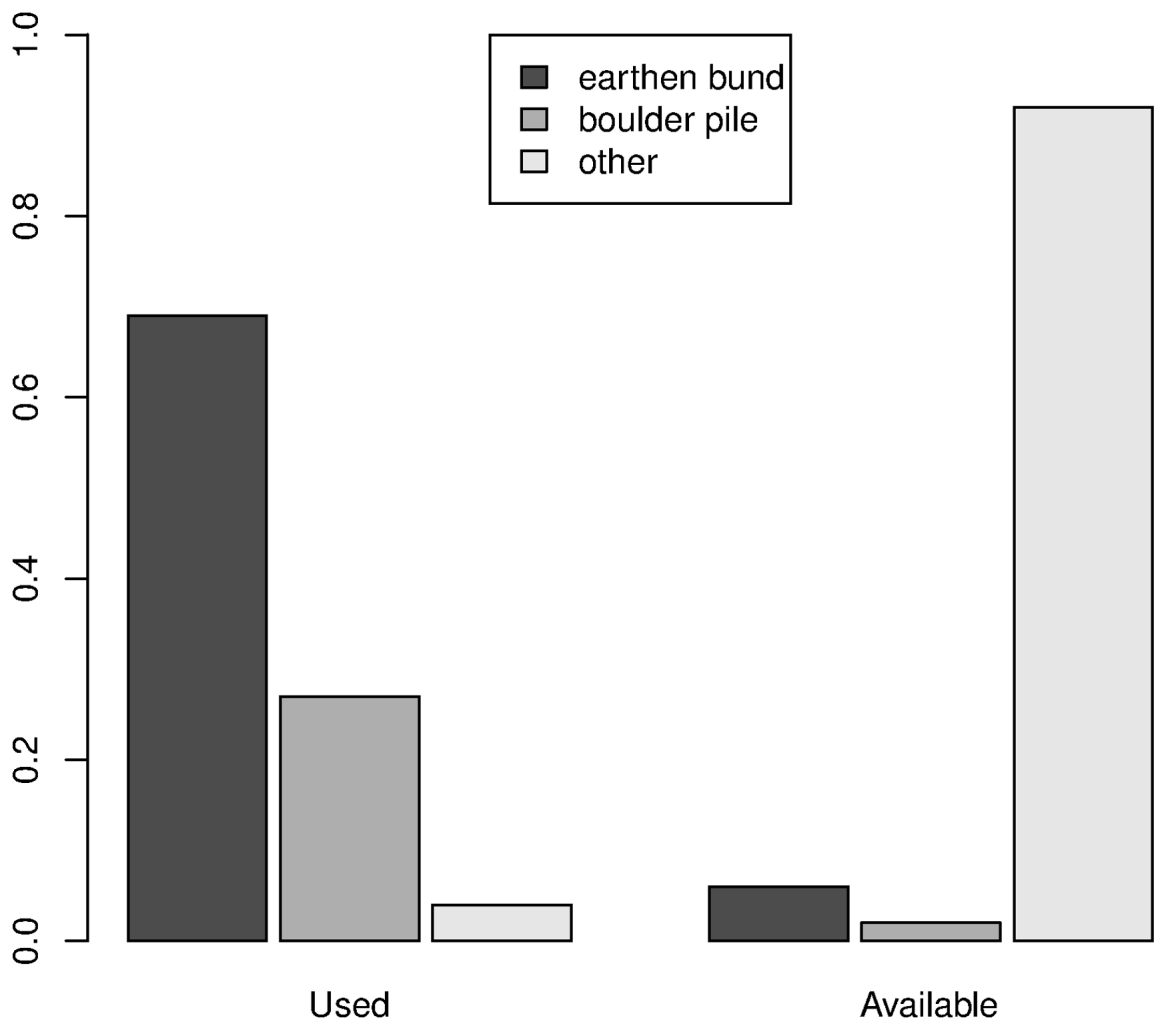
Den substrates in used and available sites. Figure shows proportion of dens found in earthen bunds, boulder piles, and other categories, as compared to proportion of substrates at putatively available sites at the den-area scale.

At the den-area scale, we used a total of 130 data points (26 dens and 104 available points). Den substrate was excluded as a variable in the models, since it was causing convergence issues in model fitting, likely due to quasi-complete separation of the categorical variable in used and available sites. Most dens occurred in earthen bunds (69%) and boulder piles (27%), while most available sites (92%) comprised of other substrates. Proportion of dens found in earthen bunds, boulder piles, and other substrates, as compared to proportion of den substrates at putatively available sites are shown in Figure 3.

Three of the eleven models used at the smaller scale received most support (Δ AICc < 4, Table 2). Rodent abundance and visibility were the most important predictors for selection of den-sites at the smaller scale (Table 3). When the choice set included rodent abundance and visibility, the odds of selecting a den-site increased by eight and four times respectively. Although shrub density and the interaction effect of rodent abundance and visibility were included in the top models, they did not seem important predictors due to high variance in the estimates (95% CI of odds ratio included 1). K-fold cross validation results showed that all the top models correctly predicted sites used by foxes 89% of the time (K- fold cross validation prediction error (delta) = 0.11 for top three models, when K = 1 and K=10).

**Table 2:** Table shows the list of models ranked by AICc explaining breeding den-site selection of Indian foxes (*V. bengalensis*) at the den-area scale.

**Model**	**K**	**AICc**	**ΔAICc**	***w_i_***
^a^ no. rodent_b + visibility*	3	47.48	0.00	0.40
no. rodent_b + shrub_den + visibility*	4	48.08	0.60	0.30
no. rodent_b + visibility + no. rodent_b *X* visibility*	4	48.30	0.82	0.27
no. rodent_b	2	53.95	6.47	0.015
no. rodent_b + shrub_den	3	54.55	7.07	0.01
no. rodent_b + shrub_den + no. rodent_b *X* shrub_den	4	55.43	7.95	<0.01
shrub_den + visibility + shrub_den *X* visibility	4	78.04	30.56	<0.01
shrub_den + visibility	3	81.00	33.52	<0.01
visibility	2	82.16	34.68	<0.01
shrub_den	2	82.71	35.23	<0.01

K = number of parameters; AICc = Akaike’s information criterion corrected for small sample sizes; ΔAICc = Delta AICc; *w*_*i*_ = Akaike’s weight

a no. rodent_b = number of rodent burrows; visibility = Visibility; shrub_den = shrub density; *X* = interaction term

* indicates top models from the candidate model set

**Table 3:** Table shows model-averaged parameter estimates and associated odds ratios from top models used at the den-area scale.

**^a^ Variable**	**Wt. Beta**	**Un.SE**	**Odds ratio**	**Lower CI**	**Upper CI**
**no. rodent_b**	**2.09**	**0.57**	**8.07**	**2.68**	**24.30**
**visibility**	**1.40**	**0.65**	**4.05**	**1.15**	**14.27**
shrub_den	0.06	0.09	1.06	0.88	1.27
no. rodent_b *X* visibility	0.07	0.13	1.07	0.83	1.37

a no. rodent_b = number of rodent burrows; visibility = Visibility; shrub_den = shrub density; *X* = interaction term

Wt. Beta = Weighted parameter estimates; Un.SE = Unconditional Standard errors; CI = 95% confidence interval

## Discussion

Indian foxes selected sites for denning closer to native grasslands at the larger home-range scale, and showed no significant selection or avoidance of any other habitat type. This finding is consistent with that of Vanak and Gompper [8], who concluded that the spatial ecology of Indian foxes is largely influenced by the presence of native grasslands in this human-modified landscape. Similar selectivity for denning in native habitats has been documented in previous research on swift foxes (*V. velox*) in northwestern Texas, which indicated a specialization for native short-grass prairies, and a complete avoidance of irrigated agricultural fields [35]. Studies on Pampas foxes (*Pseudalopex gymnocercus*) also showed a preference for open native grassland areas in South America [36,37]. Although, we did not find avoidance of agriculture or plantation habitats by Indian foxes when selecting den sites, it is noteworthy that the mean of the distance ratios for both habitats at the home-range scale were above 1, suggesting avoidance to some extent.

We also found that Indian foxes den closer to roads than random at the home-range scale. This result, although contrary to our expectation, could indicate that proximity to roads offers potential hunting or scavenging opportunities to denning foxes. Although, Indian foxes seem to avoid human-derived food sources [21], they may opportunistically scavenge road-killed reptiles, invertebrates, and rodents during the night, when traffic intensity on roads is lower. Foxes may also den closer to roads than expected so as to potentially avoid sympatric canids, the Indian wolf (*Canis lupus pallipes*) and golden jackal (*C. aureus*), that may not be as tolerant of human-associated features. Similar avoidance was observed in denning red foxes towards coyotes in north Dakota and east-central Illinois, USA [38,39]. However, this is entirely speculative, and the effect of roads on Indian fox survival needs to be examined further. In a study on island foxes (*Urocyon littoralis*), Snow et al. [40] found lower annual survival (0.76) in individuals living near road habitats, as opposed to those in the interiors (0.97).

At the smaller den-area scale, microhabitat characteristics such as rodent abundance, visibility, and the presence of human-made den substrates, determined selection of den-sites. Small rodents form a considerable part (*c.* 20% relative occurrence) of Indian fox diet [21,22]. Therefore, distribution and abundance of rodents should influence selection of breeding den sites, when reproductive costs are high. Though invertebrates are the most dominant component in the diet of the species (*c.* 33% relative occurrence), we recognize our limitation as we were unable to quantify them in different habitats in this study. However, we expected invertebrate abundance to be much lower in agricultural areas due to the use of pesticides. Given that most dens were found in grasslands, invertebrates were possibly most abundant in grassland habitats as opposed to others. Distribution of important food sources, lemmings and snow goose (*Chen caerulescens atlantica*) colonies, was found to influence selection of den sites in Arctic foxes (*Vulpes lagopus*) in Canada [41]. Small rodents may also act as den providers for the Indian fox, which may expand an existing network of rodent burrows (especially *Tatera indica* colonies), and thereby reduce energetic costs of digging dens [12]. Other species such as the swift fox and kit fox (*V. macrotis*) have similarly been associated with den providers, such as badgers (*Taxidea taxus*) and prairie dogs (*Cynomys* spp.) [42].

High visibility at den-sites should functionally reduce predation risk for vulnerable pups [42]. When selecting den sites, Indian foxes seem to avoid areas where predator detection is poor. This is not surprising given that dogs are major interference competitors of foxes in this landscape [20]. Additionally, fox pups may also be vulnerable to intraguild killing from the Indian wolf and golden jackal which are known to appropriate and use Indian fox dens for breeding [10,12,43]. Both swift foxes in prairie grasslands of northern Texas, and San Joaquin kit foxes (*V. m. mutica*) in central California, USA, avoided habitats with low visibility and high vulnerability to coyote predation [35,44]. In South Africa, cape foxes (*V. chama*), and bat-eared foxes (*Otocyon megalotis*) also avoided bushveld habitat when denning, likely due to poor visibility of black-backed jackals (*C. mesomelas*), predators of young foxes [45]. Visibility and vulnerability to predation also likely determine why Indian foxes choose short grasslands over forestry plantations or agricultural areas for denning.

Lastly, in our study, we found that Indian foxes opportunistically chose human-made structures such as earthen bunds, or boulder piles over other substrates when selecting den sites. Indian foxes have been noted to dig complex dens in areas with suitable natural substrates [12,13,16]. Chosen substrates likely offer suitable soil depth, but we were unable to measure soil depth in this study. Habib and Kumar [43], who examined den shifting by wolves in the same landscape, noted that the substrate in the region is rocky; therefore human-made structures likely offer suitable soil depth and slope to Indian foxes, which facilitate den digging. Behavioural flexibility in den-use has also been noted in San Joaquin kit [46] and Arctic foxes [47] in human-modified areas. Flexibility in using artificial substrates may benefit some species by increasing their chances of reproduction, especially where natural substrates are a limitation.

## Conclusions

During the study, the majority (*c.* 23%) of short grassland habitats in the area were communal and private landholdings, with a high vulnerability to anthropogenic modification. Protected grasslands, though present, comprised few disjunct portions and formed a very small part (1.3%) of the total study area. Grasslands in India have been categorized as ‘neglected ecosystems’, since unprotected grassland habitats, which are often communal grazing lands have been extensively converted to agriculture and diverted for industrial purposes [7]. Majority of grassland habitats now remain in a matrix of human-modified land-use; therefore, conserving and effectively managing remnant grasslands within multiple-use landscapes would benefit species such as the Indian fox. Our study reaffirms that the Indian fox is a grassland specialist, and we suggest future studies explore the effects of grassland fragmentation and connectivity on survival rates of Indian foxes. A useful management strategy benefiting den-dependent species in the region would be to retain human-made structures such as earthen bunds within grassland habitats. Some such human interventions, even though not intended for wildlife management, may aid in continued survival of such species in human-modified landscapes.
